# Supplementary material for: Extinction debt in local habitats: quantifying the roles of random drift, immigration and emigration
Source: R Soc Open Sci. 2020 Jan 15;7(1):191039. doi: 10.1098/rsos.191039 (PMC7029950; doi:10.1098/rsos.191039)
Supplement: Additional Methods [file rsos191039supp1.docx]

**ESM file 1**

**Extinction debt in local habitats: quantifying the roles of random drift, immigration, and emigration**

Additional Methods

*A full immigration model*

The full immigration model is derived from previous literature [1,2] and can be described by the following ordinary differential equation system:

$\left\{\begin{matrix} \frac{dp_{0}(t)}{dt}=p_{1}(t)-vp_{0}(t) \\ \frac{dp_{1}(t)}{dt}=vp_{0}(t)+2p_{2}(t)-(2\text{+}v)p_{1}(t) \\ \vdots\\ \frac{dp_{j}(t)}{dt}=(j+1)p_{j+1}(t)+((j-1)+v)p_{j-1}(t)-(2j+v)p_{j}(t) \end{matrix}\right.$ (S1)

The initial condition is $p_{n}(0)=\delta(n-j)$ ($j\geq0$), where the function, $\delta$, is defined in the main text. A flowchart for showing the transition dynamics of neighboring abundance states can be seen in Fig. S1B. Note that this full immigration model does not take account of the role of emigration, but it has a strong dependency between higher abundance state and immigration, as immigration will enter those terms when the abundance state increases (Eq. S1 and Fig. S1B). To this end, we can compare it to our original model (Eq. 1 of the main text) for evaluating potential impacts of higher abundance state-dependent immigration process on species extinction.

The associated moment generating function can be derived from previous literature [1,2], and accordingly, the probability of extinction or absence, by assuming that both birth and death rates are equal to 1 as the model used in the main text (see Table 1 of the main text), can be derived as

$$p_{0}\left( t \right)=\frac{t^{j}}{\left( 1+t \right)^{j+v}}.$$

As a consequence, the associated species loss model can be formulated as

$$E_{new}\left( t | A \right)=\sum_{j=1}^{\infty} S_{j}\left( 0 | A \right)p_{0}\left( t \right)=\sum_{j=1}^{\infty} S_{j}\left( 0 | A \right)\frac{t^{j}}{\left( 1+t \right)^{j+v}}.$$

The performance of the species loss model using the full immigration model $E_{new}\left( t | A \right)$ actually is quite close to the one used in the main text (Eq. 8 of the main text), as the curve shapes were nearly matched (Figs. 1, 2, and 4 in the main text). However, the difference is expected, which can be explained by two facts: 1) the full immigration model ($E_{new}\left( t | A \right)$) compared here does not take account of the emigration effect, while the original model (Eq. 8 of the main text) can incorporate both emigration and immigration effects simultaneously; 2) the full immigration model allows the contribution of immigration on the transition dynamics of large abundance states (Fig. S1B), while the original model allows the flow of immigration when species is totally absent from the target area.

*The time-dependent solution of total biodiversity loss, regardless of at the metacommunity level (Eq. 9 in the main text) or at the local area level, is a concave function when both the immigration and emigration rates are zero; however, it is indeterministic when the immigration rate is zero and the emigration rate is nonzero*

To prove this, we use Eq. 9 in the main text as an example,

$$E\left( t | A \right)=\left\{ \begin{aligned} \sum_{j=1}^{\infty} S_{j}\left( 0 | A \right)\left( \frac{t}{1+t} \right)^{j}, u=0; \\ \sum_{j=1}^{\infty} S_{j}\left( 0 | A \right)\left( \frac{1-e^{-ut}}{1-{(1-u)e}^{-ut}} \right)^{j}, u>0. \end{aligned} \right.$$

When *u* = 0, the first and second derivatives of $E\left( t | A \right)$ are

$$E^{'}\left( t | A \right)=\sum_{j=1}^{\infty} S_{j}\left( 0 | A \right)\left( \frac{t}{1+t} \right)^{j-1}j\left( \frac{1}{1+t}-\frac{t}{\left( 1+t \right)^{2}} \right)=\sum_{j=1}^{\infty} S_{j}\left( 0 | A \right)\left( \frac{t}{1+t} \right)^{j-1}j\frac{1}{\left( 1+t \right)^{2}}=\sum_{j=1}^{\infty} S_{j}\left( 0 | A \right)j\frac{t^{j-1}}{\left( 1+t \right)^{j+1}}$$

and

$$E^{''}\left( t | A \right)=\frac{d}{dt}\sum_{j=1}^{\infty} S_{j}\left( 0 | A \right)j\frac{t^{j-1}}{\left( 1+t \right)^{j+1}}=\sum_{j=1}^{\infty} S_{j}\left( 0 | A \right)j\frac{\left( j-1 \right)t^{j-2}}{\left( 1+t \right)^{j+2}},$$

respectively. Since $E^{''}\left( t | A \right)$ is always positive, we prove that $E(t|A)$ is a convex function of *t*.

Note that the case for *u* > 0 follows a similar derivation as for $u=0$; we found the curvilinear pattern of $E\left( t | A \right)$ is dependent on time *t*. Specifically, the second derivation of $E\left( t | A \right)$ is shown to have an analytical form as follows:

$$E^{''}\left( t | A \right)=\sum_{j=1}^{\infty} S_{j}\left( 0 | A \right)ju^{2}(u\left( 1-u \right)e^{-2ut}+ju^{2}e^{-ut}-u)\frac{e^{-ut}\left( 1-e^{-ut} \right)^{j-2}}{\left[ 1-\left( 1-u \right)e^{-ut} \right]^{j+2}} ,$$

from which, it reveals that the curvilinear pattern of $E\left( t | A \right)$ is indeterministic.

*The time-dependent solution of species richness, equation 4 presented in Sgardeli et al.* [3]*’s paper, is a concave function in most cases in the study of extinction debt*

To prove this, we firstly write down equation 4 presented in Sgardeli *et al.* [3]’s paper as

$$S\left( t \right)=S_{eq}+\frac{2S_{eq}}{\frac{S_{0}+S_{eq}}{S_{0}-S_{eq}}e^{rt}-1}, \left( S2 \right)$$

where $S_{eq}=J\sqrt{\frac{w}{w+b}}$, *J* is the community size, $S_{0}$ is the initial community species richness, *w* is the speciation rate and $u$ is the birth rate. Moreover,$r=2w/S_{eq}$ represents the relaxation rate in Sgardeli *et al.*’s paper. To specifically demonstrate that this is a convex function with respect to $t>0$, one can see that the first derivative of Eq. S2 with respect to *t* is

$$S^{'}\left( t \right)=-\frac{2S_{eq}r\frac{S_{0}+S_{eq}}{S_{0}-S_{eq}}}{\left( \frac{S_{0}+S_{eq}}{S_{0}-S_{eq}}e^{rt}-1 \right)^{2}}.$$

Therefore, one can see $S'(t)$ is always negative; meanwhile $S'(t)$ is increasing with *t* if $S_{0}>S_{eq}$, this is true in most cases based on the previous literature on extinction debt (and also in our paper) [4–8]. To this end, it can be concluded that Sgardeli *et al.* ’s main equation is convex in most cases.

*Comparison between the proposed species loss model (Eq. 8 in the main text) and Sgardeli et al.-derived model*

As shown above in Eq. S2, Sgardeli *et al.*-derived species loss model can be formulated as

$$E_{S}\left( t \right)=S_{0}-S\left( t \right)=S_{0}-S_{eq}-\frac{2S_{eq}}{\frac{S_{0}+S_{eq}}{S_{0}-S_{eq}}e^{rt}-1}. (S3)$$

In most cases, because $S\left( t \right)$ is convex, $E_{S}\left( t \right)$ becomes concave. Moreover, we re-write $E_{S}\left( t \right)$ as an explicit function of speciation rate *w* as

$$E_{S}\left( t \right)=S_{0}-S_{eq}-\frac{2S_{eq}}{\frac{S_{0}+S_{eq}}{S_{0}-S_{eq}}e^{\frac{2w}{S_{eq}t}}-1}. (S4)$$

This equation shows that $E_{S}\left( t \right)$ is only related to three parameters *w*, $S_{0}$ and $S_{eq}$ (community size *J* can be controlled using $S_{eq}$, and thus its influence is not necessarily discussed here). The parameter *w* has similar roles with respect to the immigration rate *v* in our paper, and $S_{0}$ is also required in our model when modeling species loss over time (Eq. 8 of the main text). $S_{eq}$ does not have a comparable parameter with respect to Eq. 8 of our present study, and this can mean that it can be used flexibly to adjust the curvilinear pattern in Sgardeli *et al.*-derived model. Therefore, we simulate Eq. S4 for comparing its temporal trajectory with respect to that of our model (Eq. 8 of the main text). Fig. S4 showed that both models actually can be similar in terms of the curve shape. However, the loss magnitude and the changing rate of the curves varied (Fig. S4).

REFERENCES

1. Allen L. 2010 *An introduction to stochastic processes with applications to biology*. 2nd edition. Boca Raton, FL: Chapman and Hall/CRC.

2. Bailey N. 1990 *The elements of stochastic processes with applications to the natural science*. New York, NY: John Wiley & Sons.

3. Sgardeli V, Iwasa Y, Varvoglis H, Halley J. 2017 A forecast for extinction debt in the presence of speciation. *J. Theor. Biol.* **415**, 48–52.

4. Halley J, Sgardeli V, Triantis K. 2014 Extinction debt and the species-area relationship: a neutral perspective. *Glob. Ecol. Biogeogr.* **23**, 113–123.

5. Halley J, Iwasa Y. 2011 Neutral theory as a predictor of avifaunal extinctions after habitat loss. *PNAS* **108**, 2316–2321.

6. Tilman D, May RM, Lehman CL, Nowak MA. 1994 Habitat destruction and the extinction debt. *Nature* **371**, 65–66. (doi:10.1038/371065a0)

7. Wearn O, Reuman D, Ewers R. 2012 Extinction Debt and Windows of Conservation Opportunity in the Brazilian Amazon. *Science* **337**, 228–232.

8. Veresoglou S, Halley J, Rillig M. 2015 Extinction risk of soil biota. *Nat. Commun.* **6**, 8862.
