# Supplementary material for: Extinction debt in local habitats: quantifying the roles of random drift, immigration and emigration
Source: R Soc Open Sci. 2020 Jan 15;7(1):191039. doi: 10.1098/rsos.191039 (PMC7029950; doi:10.1098/rsos.191039)

**ESM file 2**

**Extinction debt in local habitats: quantifying the roles of random drift, immigration, and emigration**

**Additional Figures**

Fig. S1. A flowchart for showing the transition dynamic between neighboring abundance states for the stochastic models studied in the paper. Subplot A represents Eq. 1 of the main text and subplot B represents Eq. S1 of the Appendix.

A)


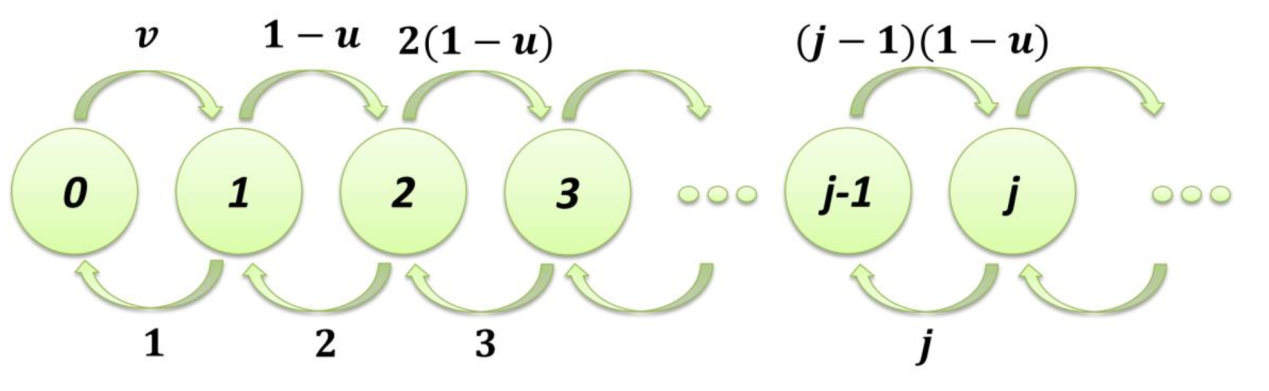


B)


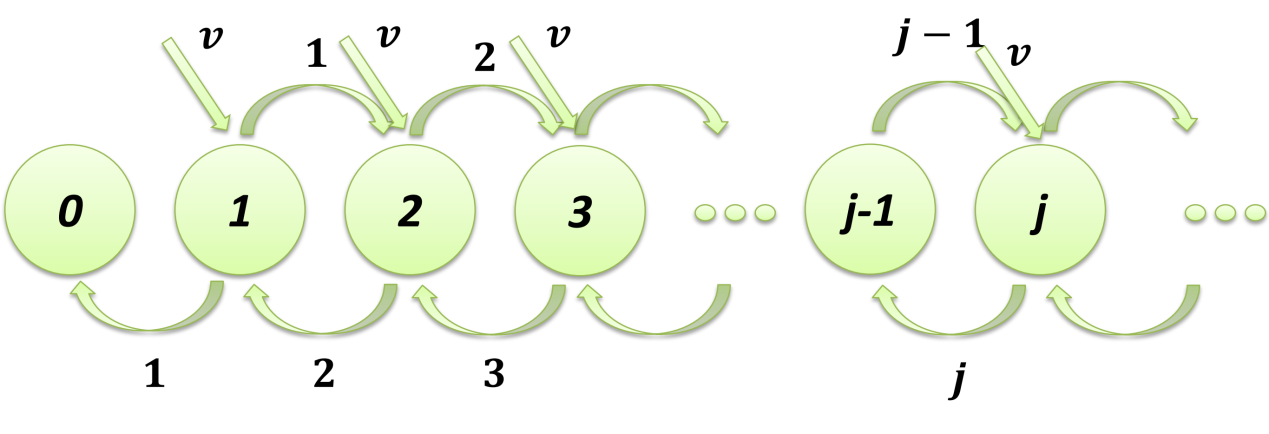


Fig. S2. Impacts of initial abundance state on the resultant extinction probability over time. All the simulations have assumed the immigration rate *v* = 0.01, while the emigration rate *u* = 0.000001. Three initial abundance states (3, 5, and 7) are considered and compared. The time interval between two adjacent time-steps is set to 0.002.


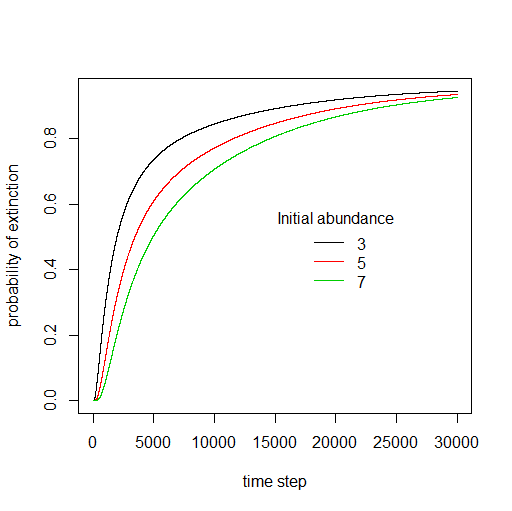


Fig. S3. Impacts of different combinations of emigration and immigration rates on the resultant extinction probability over time. All the simulations have set the initial abundance state to 3 and the time interval between two adjacent time-steps as 0.002.


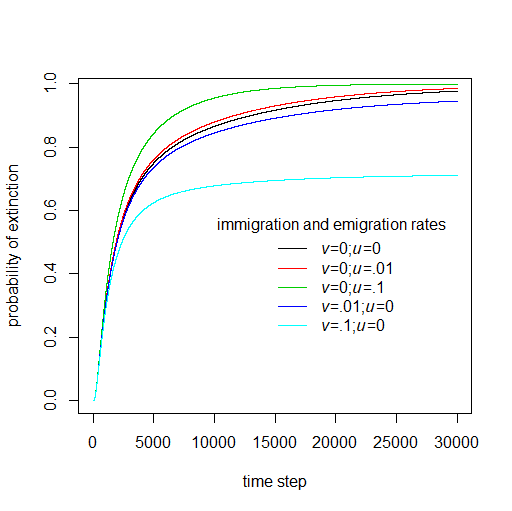


Fig. S4. Similarity of the proposed biodiversity loss models used in the present study (Eq. 8 of the main text) and the previous study (Sgardeli et al. [3]). Both the immigration rate *v* in our model and the speciation rate *w* in Sgardeli et al.’s model are assumed to be the same as 0.02. Initial species richness is also the same as 1000. Additionally, $S_{eq}=40$ was used in Sgardeli et al.'s paper while parameter $\omega$ in Fisher’s logseries model was set to 0.1 in our model. The y-axis denotes the difference on the number of species lost at a specific time with respect to the original species number at the initial time.


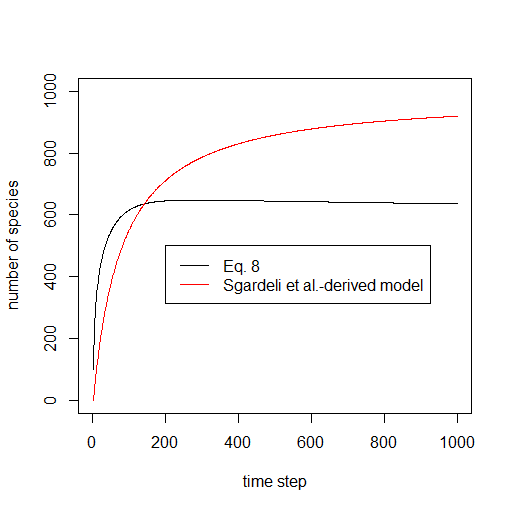


Fig. S5. The influence of parameter omega ($\omega$) on determining the initial species abundance distribution and the likelihood for a species to be absent from the local target community in the area-based logseries model. When the omega value is higher, the probability of being absent from the local area is higher; thus, the expected species richness in the local area tends to be smaller (after lumping the probabilities of the presence of all species). The entire region has a total areal size of *A* = 100, while the size of the local intact area of interest was set to *a* = 10.


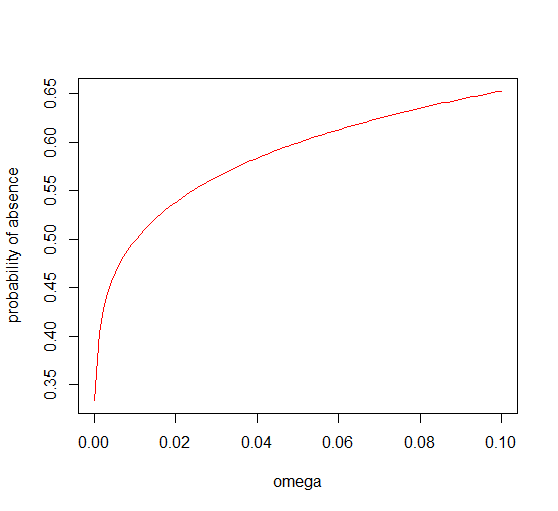


Fig. S6. A shift in the emigration rate (*u*) at the time point of habitat destruction and its influence on the magnitude of the extinction debt or species richness over time. The entire region has a total areal size of *A* = 100. The areal size of the destroyed habitat was set to *b* = 50 while the local intact area of interest was set to a size of *a* = 10. Parameter $\omega$ in Fisher’s logseries model was set to 0.001. The species richness increased to some extent at the time point of habitat destruction (marked as a vertical gray dashed line).


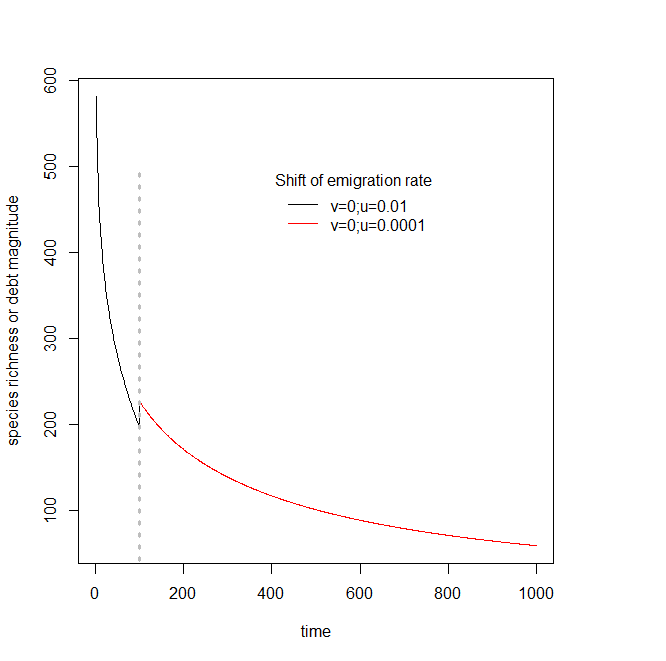

Supplement: Additional Figures [file rsos191039supp2.docx]
